# Pediatric Burn Survivors Have Long-Term Immune Dysfunction With Diminished Vaccine Response

**DOI:** 10.3389/fimmu.2020.01481

**Published:** 2020-07-21

**Authors:** Blair Z. Johnson, Sonia McAlister, Helen M. McGuire, Vetrichevvel Palanivelu, Andrew Stevenson, Peter Richmond, Debra J. Palmer, Jessica Metcalfe, Susan L. Prescott, Fiona M. Wood, Barbara Fazekas de St Groth, Matthew D. Linden, Mark W. Fear, Vanessa S. Fear

**Affiliations:** ^1^School of Biomedical Sciences, The University of Western Australia, Perth, WA, Australia; ^2^School of Medicine, The University of Western Australia, Perth, WA, Australia; ^3^Wesfarmers Centre of Vaccines and Infectious Diseases, Telethon Kids Institute, Perth, WA, Australia; ^4^Ramaciotti Facility for Human Systems Biology and the Charles Perkins Centre, Discipline of Pathology, The University of Sydney, Sydney, NSW, Australia; ^5^Centre for Allergy and Immunology Research, Telethon Kids Institute, Perth, WA, Australia; ^6^Department of Health WA, Perth, WA, Australia; ^7^Genetic and Rare Diseases, Telethon Kids Institute, Perth, WA, Australia

**Keywords:** non-severe burn injury, immunity, vaccination, mass cytometry, acute trauma, systemic

## Abstract

Epidemiological studies have demonstrated that survivors of acute burn trauma are at long-term increased risk of developing a range of morbidities. The mechanisms underlying this increased risk remain unknown. This study aimed to determine whether burn injury leads to sustained immune dysfunction that may underpin long-term morbidity. Plasma and peripheral blood mononuclear cells were collected from 36 pediatric burn survivors >3 years after a non-severe burn injury (<10% total body surface area) and from age/sex-matched non-injured controls. Circulating cytokine and vaccine antibody levels were assessed using multiplex immunoassays and cell profiles compared using a panel of 40 metal-conjugated antibodies and mass cytometry. TNF-α (1.31-fold change from controls), IL-2 (1.18-fold), IL-7 (1.63-fold), and IFN-γ (1.18-fold) were all significantly elevated in the burn cohort. Additionally, burn survivors demonstrated diminished antibody responses to the diphtheria, tetanus, and pertussis vaccine antigens. Comparisons between groups using unsupervised clustering identified differences in proportions of clusters within T-cells, B-cells and myeloid cells. Manual gating confirmed increased memory T-regulatory and central memory CD4+ T-cells, with altered expression of T-cell, B-cell, and dendritic cell markers. Conclusions: This study demonstrates a lasting change to the immune profile of pediatric burn survivors, and highlights the need for further research into post-burn immune suppression and regulation.

## Introduction

Burns continue to impact the lives of millions of people each year; from new injuries to ongoing recovery, the psychological, physical, and financial burden is persistent. In 2004 the World Health Organization (WHO) estimated that 11 million people globally required medical attention for a burn injury (1). A more recent annual report from the Burns Registry of Australia and New Zealand (BRANZ) recorded 3,295 cases treated at specialized burn clinics across the two countries (2016–2017), with pediatric cases accounting for 30% of the cohort (2).

Patient outcomes are influenced by the severity of the burn injury (3, 4). Total body surface area (TBSA) involvement is used to classify burns as severe (≥20% TBSA) or non-severe (<20% TBSA). Due to their profound local and systemic effects (5, 6), severe burns remain the focus of the majority of burns research. However, the majority of burns (84%) involve a TBSA of <10% (2), and it is becoming increasingly apparent that even non-severe burns have a long-term impact on the health of survivors.

Epidemiological studies have found that burn survivors, regardless of severity, are at increased risk for a range of diseases even decades after injury, and typically have a longer length of stay when hospitalized for them. These include cardiovascular diseases (7, 8), nervous disorders (9), musculoskeletal diseases (notably infectious and inflammatory polyarthropathies) (10), cancers (11), diabetes mellitus (12), gastrointestinal diseases (13), and infections (14). Extensive data in the literature support a role for innate and adaptive immune cell dysfunction in the pathogenesis of the diseases that have an elevated incidence in burn survivors (15–19), suggesting immune dysfunction may contribute to post-burn morbidity.

In our laboratory pre-clinical studies in mice, modeling 8% TBSA involvement as a non-severe burn injury (NSBI), have demonstrated changes in innate and adaptive immunity up to 84 days post-injury (14, 20). In pediatric patients with severe burn injury, sustained elevation of circulating cytokines has been observed up to 3 years after the injury (21). In this study we have investigated whether there is an enduring change within the immune compartment of pediatric patients more than 3 years after a non-severe burn injury. We hypothesized that patients would manifest significant changes to the circulating immune profile compared to uninjured controls, reflecting a sustained impact of acute but non-severe burn trauma on the immune system.

## Materials and Methods

### Specimen Collection

Children were recruited at least 3 years after presenting for a non-severe burn injury at Princess Margaret Hospital. They were aged 0–4 years of age at the time of original presentation. Age/sex-matched controls were selected from a pool of healthy donors. All samples were obtained with informed consent of a parent or guardian and the collection was conducted with ethical approval from the Child and Adolescent Health Service WA (approval numbers: 2015219EP; 1111EP; 768EP). All patients recruited had no history of pre-existing illness and were not currently on medication at time of sampling. No patients had visible signs or recent history of acute infection at the time of blood collection. Blood was collected into tubes containing preservative-free heparin, then centrifuged to collect the plasma. The remaining blood was resuspended in RPMI-1640 (Gibco, USA) and the peripheral blood mononuclear cells (PBMCs) were isolated using Lymphoprep (STEMCELL Technologies, Canada), and then cryopreserved in 10% DMSO upon slow freeze for storage.

### Multiplex Cytokine Assay

Circulating cytokines were assessed using a customized Milliplex MAP human high sensitivity T cell panel multiplex bead assay (Merck). Cytokines tested were Tumor necrosis factor-alpha (TNF-α), Interleukin-8 (IL-8), Interleukin 7 (IL-7), Interleukin-6 (IL-6), Interleukin-5 (IL-5), Interleukin-2 (IL-2), Interleukin-1beta (IL-1β), Interleukin-17A (IL-17A), Interleukin-13 (IL-13), Interleukin-12 p70 (IL-12(p70)), Interleukin-10 (IL-10), Interferon-gamma (IFN-γ) and Granulocyte macrophage colony stimulating factor (GM-CSF). Briefly, plasma samples that had not been thawed since the original freeze were thawed, filtered using 0.45 μm syringe filters (Nalgene) and a 50 μl aliquot removed. Standards were prepared for each cytokine and plated in duplicate and assay conducted as per manufacturer's instructions. Each 96-well plate was read on a Luminex 200 instrument. Each sample was assayed in duplicate and the mean value for each cytokine/patient was used for statistical analysis.

### Diphtheria-Tetanus-Acellular Pertussis Multiplex Immunoassay

Total IgG concentrations against vaccine antigens pertussis toxin (PT), pertactin (PRN), filamentous hemagglutinin (FHA), fimbriae 2/3 (FIM 2/3), tetanus toxin (TT), and diphtheria toxoid (DT) were measured using an in-house multiplex bead-based immunoassay. PT, PRN, and FHA were kindly provided by GlaxoSmithKline (Belgium). TT was purchased from Sigma-Aldrich while DT and FIM 2/3 was sourced from List biological laboratories (USA). A standard curve was generated using a 10-step 3-fold serial dilution of an in-house reference sera previously quantified against National Institute for Biological Standards and Control reference sera: PT (06/140), TT (TE-3), and DT (10/262). The concentration of FIM 2/3 IgG was previously assigned to 06/140 by an international collaborative study (22). Blanks and two QC samples were included on every plate to calculate% critical variance across all assays, which fell between 6 and 12.7%. Assay specificity was determined using inhibition and interference assays. No cross reactivity was detected (data not shown).

The multiplex immunoassay was carried out as per van Gageldonk et al. (23), with minor modifications. In brief, Bio-Plex® COOH-microspheres (6.25 × 106) were conjugated with optimized concentrations of antigen in 1 x PBS pH 7.2 (Life Technologies, AUS) as follows: PT 10 μg/ml, PRN 75 μg/mL, FHA 25 μg/ml, FIM 2/3 100 μg/ml, TT 100 μg/mL, and DT 100 μg/ml. Samples were diluted in PBS containing 3% bovine serum albumin (BSA) and 0.05% Tween 20 (Sigma-Aldrich). MultiScreen Filter Plates (Merck) were pre-wet with 50 μl PBS containing 0.05% Tween 20 (PBS.T) and the liquid removed by vacuum manifold (2–5 mmHg). Diluted plasma samples were mixed with bead-mix (25 μl; PBS containing 4000 beads/region) in individual wells and incubated on a plate shaker (500 rpm) protected from light for 30 min. Plates were washed twice with 100 μl of 0.05% PBS.T before the addition of 100 μl 1:200 RPE-conjugated goat-anti human IgG Fc secondary antibody (Jackson ImmunoResearch Laboratories Inc.) and incubated as above for a further 30-min. Following washing, the beads were resuspended in 125 μl 0.05% PBS.T and read using a bioplex-200 machine. Antigen-specific IgG concentrations (mIU/mL) were determined using a 5-PL linear curve generated with Bioplex Manager software version 5.0.

### Immunophenotyping by Mass Cytometry

All reagents used for mass cytometry were prepared in plastics that had not been exposed to detergents, to avoid barium contamination. Stain buffer was prepared as 0.1% bovine serum albumin (Sigma-Aldrich, Australia), 2 mM EDTA (Sigma-Aldrich), and 0.05% sodium azide (Sigma-Aldrich) dissolved in calcium/magnesium-free phosphate buffered saline (PBS; Gibco) and adjusted to pH 7.4. 4% paraformaldehyde (PFA) prepared fresh each day by dissolving PFA (Sigma-Aldrich) in PBS and adjusting pH to 7.4.

Metal-labeled antibodies (Table 1) were validated, pre-tittered, and supplied in per-test amounts by the Ramaciotti Facility for Human Systems Biology Reagent Bank. Reagent bank antibodies were either purchased from Fluidigm in pre-conjugated form or unlabeled antibodies were purchased in a carrier-protein-free format and conjugated at the Ramaciotti Facility with the indicated metal isotope using the MaxPAR conjugation kit (Fluidigm, South San Francisco, CA) according to the manufacturer's protocol. Four Element EQ Beads, Maxpar water, Cell-ID cisplatin, and Cell-ID DNA intercalator were purchased from Fluidigm.

**Table 1:** Antibody cocktails for immunophenotyping PBMCs by mass cytometry.

	**Marker**	**mAb**	**Isotope label**
a) Surface stain 1	CD45^*^	HI30	Pd_104
	CD45^*^	HI30	Pd_108
	CCR2	K036C2	Eu_151
	CD183 (CXCR3)	REA232	Dy_163
	CD184 (CXCR4)	12G5	Lu_175
	CCR7	G043H7	Tb_159
b) Surface stain 2	IgD	IA6-2	Y_89
	CD11c	Bu15	In_115
	CD19	HIB19	Nd_142
	CD56	NCAM16.2	Nd_143
	CD4	RPA-T4	Nd_145
	CD8a	RPA-T8	Nd_146
	CD20	2H7	Sm_147
	CD16	3G8	Nd_148
	CD25	M-A251	Sm_149
	CD275 (ICOSL)	MIH12	Nd_150
	CD45RO	UCHL1	Gd_152
	CD68	Y1/82A	Eu_153
	CD31	WM59	Gd_155
	CD86	IT2.2	Gd_156
	CD123	6H6	Dy_161
	CCR6	11A9	Pr_141
	CX3CR1	2A9-1	Er_164
	CD61	VI-PL2	Ho_165
	CD34	581	Er_166
	CD27	M-T271	Er_167
	CD45RA	HI100	Tm_169
	CD3	UCHT1	Er_170
	CD38	HIT2	Yb_172
	CD14	M5E2	Yb_173
	HLA-DR	G46-6	Yb_174
	CCR5	HEK/1/85a	Nd_144
	CD127	A019D5	Lu_176
	CD11b	ICRF44	Bi_209
	CCR4	L291H4	Gd_158
c) Intra-cellular stain	IDO-1	700838	Gd_154
	T-bet	4B10	Gd_160
	FoxP3	PCH101	Er_162
	Ki67	B56	Er_168
	Arginase I	14D2C43	Yb_171

* *CD45 is on two isotopes in order to barcode patients (Pd_104) and controls (Pd_108). CD45 stains all leukocytes. mAb, monoclonal antibody*.

Cryopreserved PBMCs were thawed rapidly then transferred into warm RPMI + 10% heat-inactivated fetal calf serum (FCS) (Bovogen, French origin) + Pierce universal nuclease (Thermofisher, Australia). Two million live PBMCs were stained as previously described (24), with amendments: 100 μL of the first surface antibody cocktail containing an individual barcoding reagent (either Pd104-CD45 for patients or Pd108-CD45 for controls, Table 1a) was added. Samples were incubated with stain at room temperature for 30 min. Cells were washed, then patient and control pairs were combined into a single tube and washed again. The remainder of the surface staining antibodies (Table 1b) were added, 100 μL per sample, and incubated on ice for 30 min. Cells were washed twice, then permeabilized with FoxP3 Fix/Perm buffer (Thermofisher) and incubated with the intracellular antibodies (Table 1c) on ice for 30 min. Cells were fixed overnight at 4°C in 4% PFA containing 0.125 μM DNA intercalator. After washing, cells were resuspended at 8.5 × 10^5^ cells/mL in a 1:10 suspension of EQ beads and Maxpar water prior to data acquisition on a Fluidigm Helios mass cytometer.

**Figure 1:**
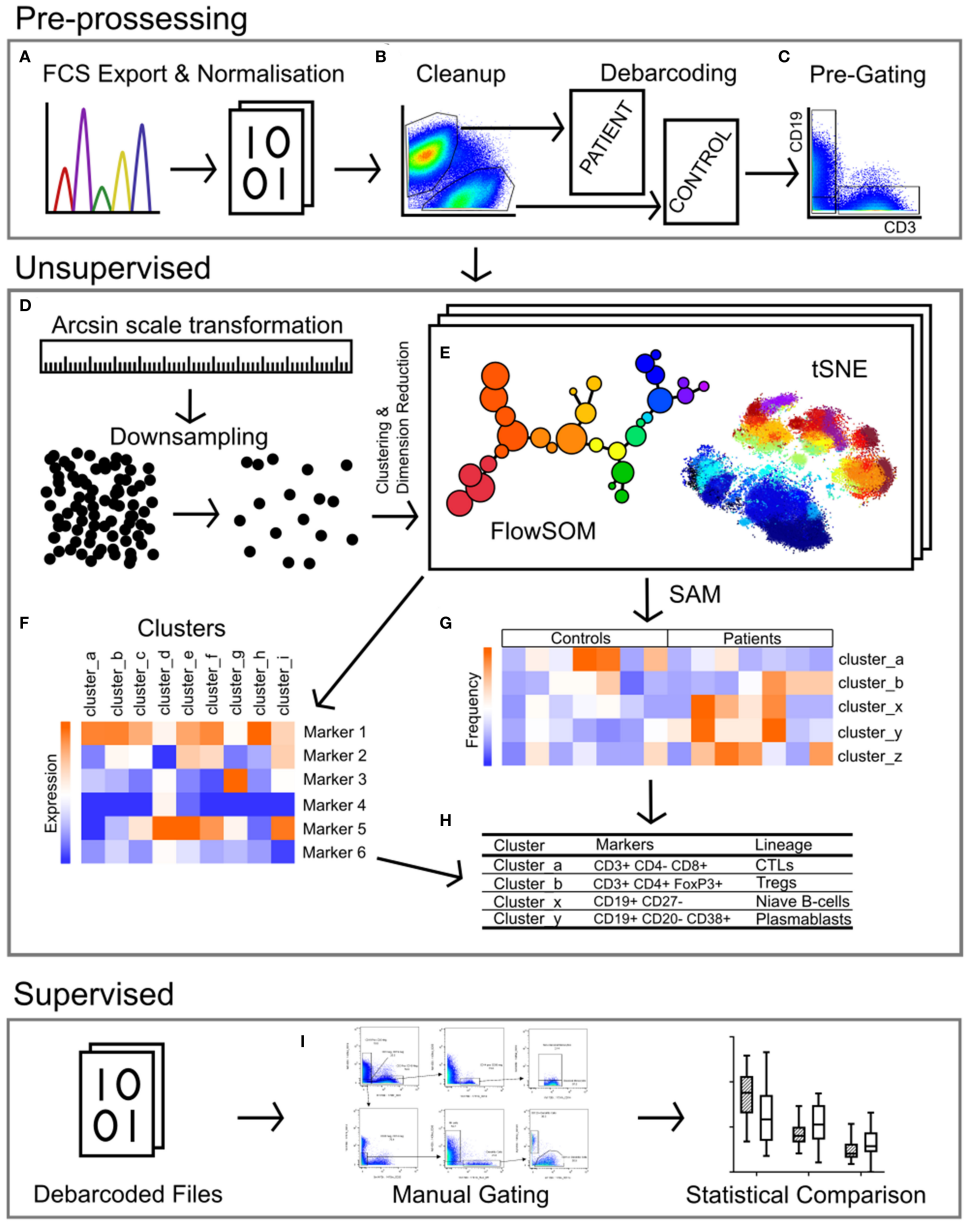
Pipeline for the analysis of mass cytometry data. **(A)** Raw files acquired from the Helios mass cytometer were normalized based on the signal intensity of bead-specific isotopes, and exported as FCS files. **(B)** Files were manually cleaned to identify intact cells based on Ir_191 DNA staining, beads removed based on bead specific Ce_140, and doublets excluded using Ir_191 DNA staining vs. event length. Patient and control samples were debarcoded and exported into separate files based on staining with CD45-Pd_104 (patients) and CD45-Pd_108 (controls). Live cells were identified based on cisplatin stain intensity. **(C)** Events were pre-gated on CD3 vs. CD19 to export files containing T-cells (CD3+ CD19-), B-cells (CD19+ CD3-) and non-T-non-B cells (CD3- CD19-, i.e., monocytes, dendritic cells, NK cells). **(D)** The signal intensity of each marker was transformed by arcsin scaling and events from each individual were downsampled then concatenated for clustering analysis. **(E)** T-cells, B-cells, and non-T-non-B-cells were clustered separately by FlowSOM and visualized by t-stochastic neighbor embedding (tSNE). This was repeated multiple times with cluster numbers ranging from 40 to 90, in order to determine the most appropriate number of clusters **(F)** based on marker expression. **(G)** Significance analysis of microarrays (SAM) was used to identify clusters with different frequencies between patients and controls **(H)**, and the lineage of these clusters was determined based on the expression of markers **(F)**. **(I)** Binary gating was employed to manually investigate previously characterized PBMC subpopulations, and to further investigate events corresponding to clusters identified in unsupervised analysis.

**Figure 2:**
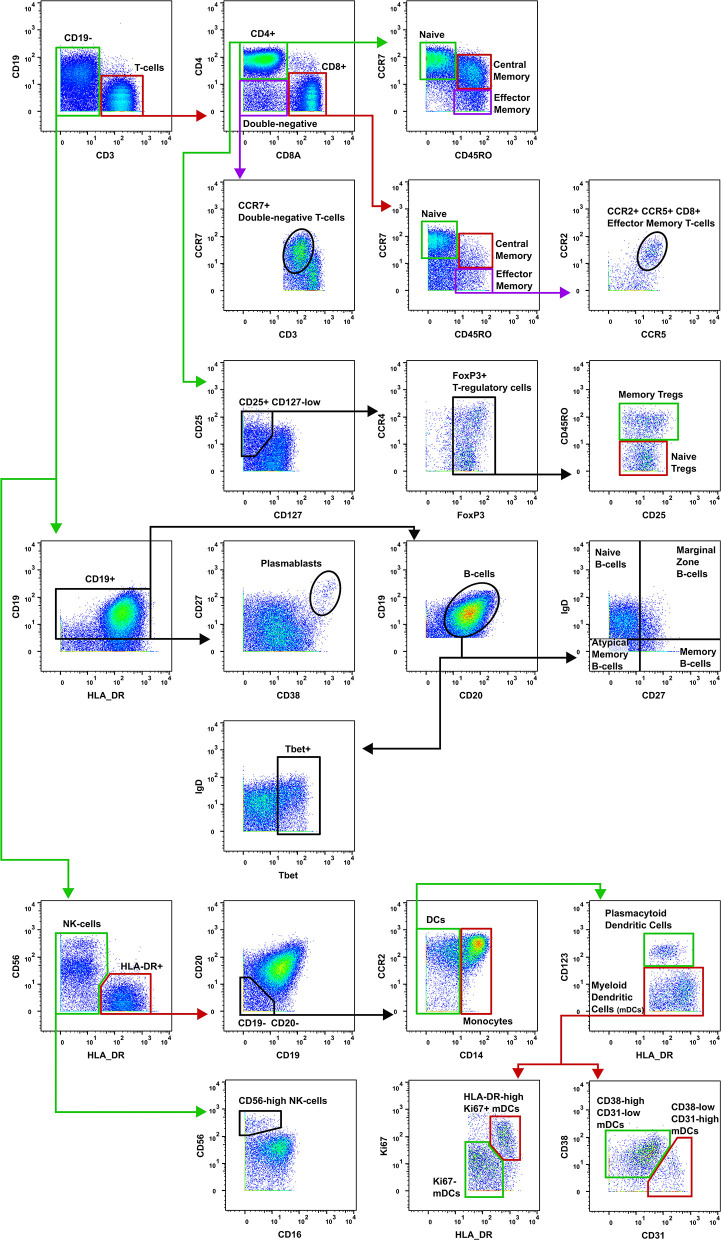
Gating strategy for T-cell subsets. The gating strategy used to quantify the frequency of T-cell subsets in burn patients and controls. CD, cluster of differentiation; Tregs, T-regulatory cells; NK, natural killer; DCs, dendritic cells; mDCs, myeloid dendritic cells.

Data were normalized using CyTOF Software (v6.7.1014, normalization passport EQ-P13H2303_ver2) (25, 26) and gated using Flowjo (v10.4.2). An overview of analysis is outlined in Figure 1. Files from each sample were cleaned by gating on Ir191_DNA, Ir193_DNA, event length, and bead-specific Ce140 to remove debris and non-cellular events. Patient and control events were debarcoded and the exported files further cleaned to remove dead cells based on high cisplatin staining (Supplementary Figure 1). T-cells (CD3+ CD19–), B-cells (CD19+ CD3–), and other cell lineages (CD3– CD19–) were gated and exported as individual files for use in downstream analysis. The CAPX data analysis pipeline (v2.5, Sydney Cytometry) (27) was then used to down-sample, transform (arcsine), cluster (FlowSOM) (28), and perform dimensionality reduction (tSNE) on the 3 gated populations for each subject. Events were clustered using all markers in each file except CD45, DNA-intercalator, cisplatin, and CD61. B-cells, T-cells, and “other cells” were analyzed with a range of cluster numbers between 10 and 90 to ensure cluster separation on meaningful markers. The intent was to identify the maximum number of clusters without artificially separating events that were biologically similar. A hierarchical gating strategy (Figure 2) was also implemented to investigate previously described PBMC subpopulations (29) and populations identified during unsupervised clustering.

### Statistics

For each sample, the mean of duplicate runs of the cytokine and vaccine antibody assays were used for Mann-Whitney comparisons of burn survivors vs. controls. Multiple Experiment Viewer (v4.9.0, TM4) (30) was used to visualize immune subset frequencies as heatmaps. Each cell in the heatmap represented the percentage of the cluster contributed by an individual subject. Significance analysis of microarrays (SAM), a technique used for large datasets (31), was implemented to identify clusters with a different frequency between paired patients and controls. Wilcoxon signed-rank test was performed on paired data from supervised gating and *p*-values adjusted for false-discovery rate. Graphs produced using GraphPad Prism (v8.0.1).

## Results

### Sample Demographics

Blood was collected from 36 pediatric burn survivors aged 4–8 years old and compared to 36 age- and sex-matched uninjured controls. Mean burn size was 3.95 ± 3.1% TBSA, mean age at time of injury was 22 months ± 9 months and mean age at time of sample collection was 6.1 years ± 1.1 years. 17 female and 19 male burn survivors were recruited (47%:53% respectively). Age at time of injury, total body surface area, etiology of injury, and time between injury and sample collection are detailed in Table 2.

**Table 2:** Details of burn injury population including age at time of injury, TBSA and etiology of the burn.

**Age at burn/months**	**TBSA (%)**	**Cause of burn**	**Time since burn to sample collection/months**
22	1	Frictional	65
13	7	Scald	59
15	3	Scald	54
18	3	Scald	51
24	<10	Scald	54
37	3	Scald	36
18	2	Frictional	44
25	2.50	Scald	52
25	8	Scald	49
18	1.5	Scald	50
16	<10	Scald	49
12	6	Scald	44
18	9	Scald	63
12	7	Scald	60
19	5	Scald	59
12	2	Chemical	65
7	1	Electrical	63
12	<2	Scald	66
18	1	Contact	62
6	<10	Sun burn	67
12	8	Scald	65
18	9	Scald	54
18	2	Thermal	55
12	1	Scald	51
18	<5	Chemical	57
12	5	Contact	53
10	<1	Contact	66
30	2	Contact	51
42	<2	Frictional	36
24	<1	Frictional	48
16	<2	Contact	55
14	3	Contact	67
41	<1	Frictional	50
38	10	Contact	59
38	1	Scald	61
36	2.50	Scald	62
36	9	Scald	54
30	<5	Frictional	63
22	1.50	Contact	60
36	1	Frictional	64
30	<1	Scald	67
36	2–3	Scald	64

The Australian National Immunisation Program recommends all children receive primary diphtheria-tetanus-acellular pertussis (DTPa) vaccinations at 6–8 weeks, 4 months and 6 months of age, followed by booster doses at 18 months and 4 years. Vaccination status was verified using the Australian Immunisation Register. Records were available for 35 of the burn survivors and 27 of the controls, confirming they had completed all DTPa vaccinations according to the Australian schedule. This included receiving the vaccination at 4 years of age, which was after the burn injury for all the burn patients. Only individuals with vaccination status records were included in the vaccine-specific antibody analysis, and the others were excluded as we could not confirm vaccination status.

### Cytokine Profiling

**Figure 3:**
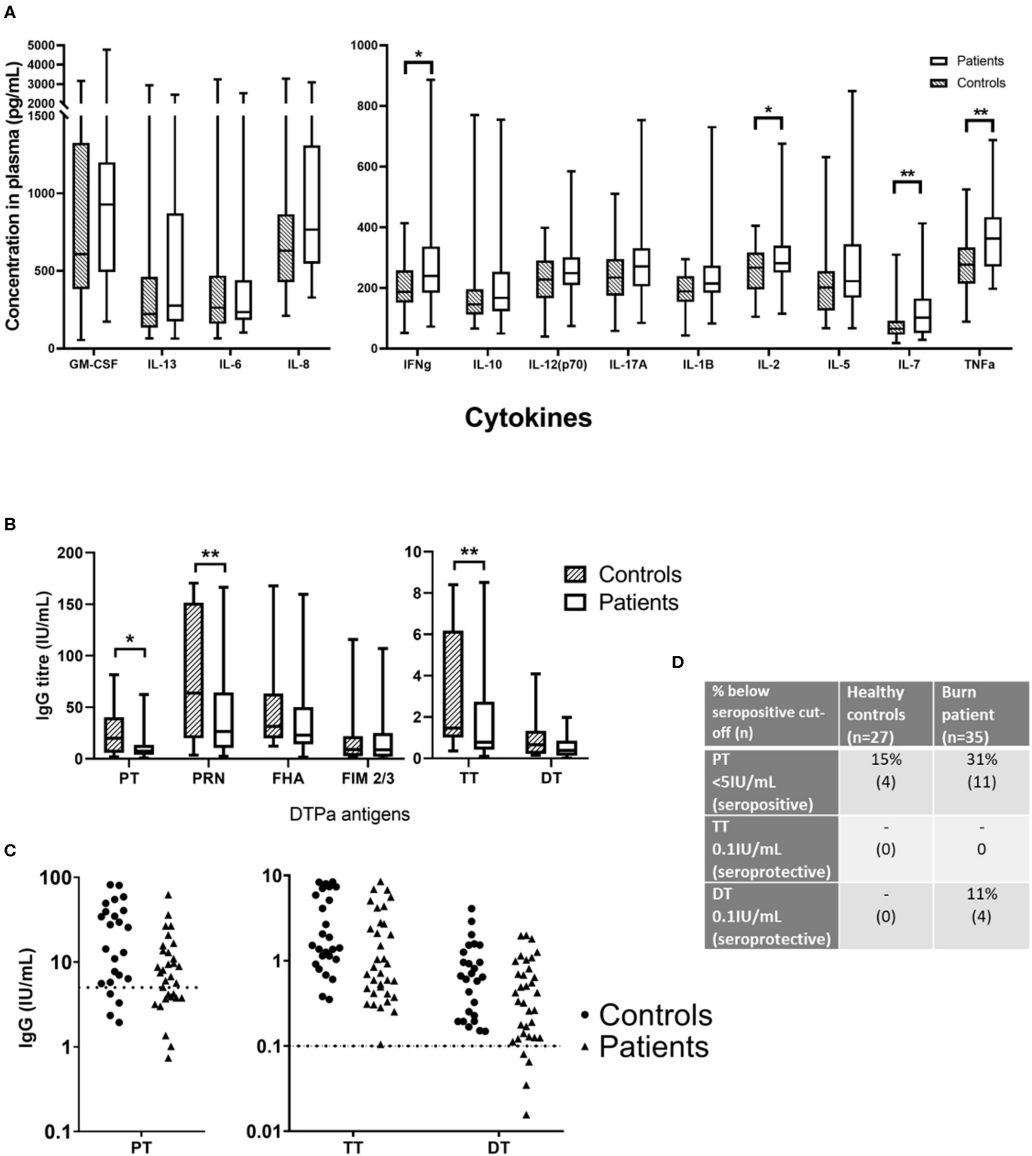
Concentrations of circulating cytokines and vaccine-specific IgG in plasma of burn survivors and controls. A multiplex cytokine assay was used to measure the concentration of 13 cytokines, and IgG targeting six antigens from the diphtheria, tetanus acellular pertussis (DTPa) vaccine. **(A)** Mann-Whitney tests used to compare burn survivors and controls (*n* = 36 age/sex-matched pairs) demonstrated four cytokines were elevated in burn survivors: interferon gamma, IL-2, IL-7, and tumor necrosis factor alpha. **(B)** IgG concentrations specific for pertussis toxin, pertactin, and tetanus toxin were lower in burn survivors; **(C)** dotted lines indicate thresholds of seropositivity (PT > 5 IU/mL, and long term seroprotection against tetanus and diphtheria (TT and DT IgG > 0.1IU/mL). **(D)** The rates of seropositivity/seroprotection in the burns cohort (*n* = 35) for pertussis toxin, tetanus toxin and diphtheria toxoid, compared to controls (*n* = 27). Experiments were performed in duplicate and the average used for analysis. **p < 0.01, *p < 0.05. GM-CSF, granulocyte-macrophage colony-stimulating factor; IL, interleukin; IFNg, interferon gamma; TNFa, tumor necrosis factor alpha; PT, pertussis toxin; PRN, pertactin; FHA, filamentous hemagglutinin; FIM 2/3, fimbriae types 2/3; TT, tetanus toxin; DT, diphtheria toxoid.

Plasma was isolated from each blood sample and tested for TNF-α, IL-8, IL-7, IL-6, IL-5, IL-2, IL-1 β, IL-17A, IL-13, IL-12(p70), IL-10, IFN-γ, and GM-CSF. Of the 13 cytokines analyzed, four were found to be significantly elevated in the burn survivors (Figure 3A). The inflammatory cytokine TNF-α was measured at a 1.31-fold concentration greater in patients compared to controls (mean ± SE controls, mean ± SE burn; *p* < 0.01). B cell and T cell modulating cytokines were also significantly increased in the burn group. Notably, IL-7 was 1.63-fold higher (*p* < 0.01), whilst IL-2 (mean ± SE con v burn) and IFN-γ (mean ± SE con v burn) both showed a 1.18-fold increase (*p* < 0.05). The elevation of these cytokines in the patient cohort suggests a sustained pro-inflammatory milieu may be present for many years after the initial acute trauma.

### Vaccine Antibodies

Antibody responses to DTPa antigens, were compared between control and burn groups in individuals who had completed the DTPa vaccination protocol according to the Australian schedule. Burn survivors showed a diminished IgG response to pertussis toxin burn mean ± SE and control mean ± SE (0.48-fold reduction, *p* < 0.05). Similarly, pertactin IgG response was significantly decreased burn mean ± SE and control mean ± SE (0.46-fold reduction, *p* < 0.01) (Figure 3B). In addition, for pertussis (PT IgG ≥ 5 IU/mL) 31% of the patient cohort was below the seropositive cut-off, compared to 15% of the controls (Figures 3C,D). A significantly diminished response in the burn group was also observed for tetanus specific IgG, burn mean ± SE and control mean ± SE (0.48-fold, *p* < 0.01). While diphtheria toxoid IgG concentrations were comparable between groups, 11% of the burn cohort were below the threshold of long-term seroprotection against diphtheria (DT IgG ≥ 0.1 IU/mL) compared with none of the controls (Figures 3C,D). This decreased response to vaccine antigens in the patient cohort, observed despite the administration of a vaccine post-injury, suggests that the acute trauma may reduce the ability to respond to vaccination, mediated by a sustained systemic change, since the vaccine was administered in many cases over a year after the injury.

### Immunophenotyping by Mass Cytometry

Of the 36 patients recruited, sufficient PBMCs were obtained from only 29 due to the small volume of blood collected. Of these 29, seven were excluded due to poor sample quality resulting from low cell viability, and two additional sample pairs were excluded as the barcoding step failed. Of the 20 remaining pairs, 13 were males and 7 were females, with a mean age of 6.3 years at time of sample collection.

**Figure 4:**
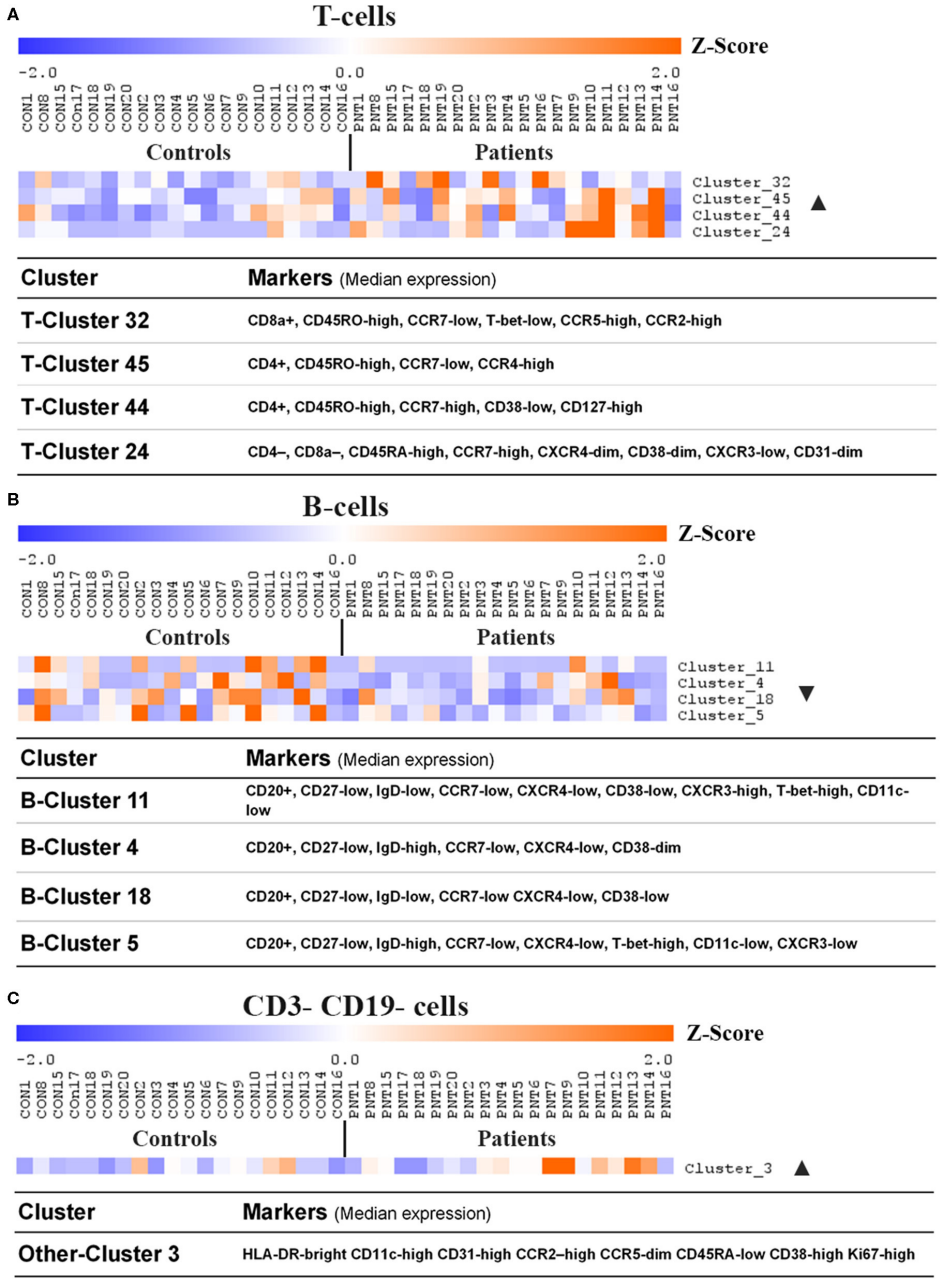
Clusters and cell lineages with disparate frequencies between burn survivors and controls determined by unsupervised clustering analysis. FlowSOM clustering was undertaken on paired data from patients and matched controls. Data was pre-gated on CD3+ and CD19+ to analyse **(A)** T-cell subpopulations, **(B)** B-cell subpopulations, and **(C)** CD3- CD19- subpopulations, respectively. Significance analysis of microarrays (SAM) was used to identify clusters with disproportionate frequencies between patients and controls, which are shown here. The frequency of events in a cluster from each individual has been normalized per-cluster (rows) and presented as a z-score. Positive and negative markers for identifying lineages were determined using median expression values of markers in each cluster. Ordered by age of pairing. n = 20 age/sex-matched pairs. CD, cluster of differentiation; ▴, increased frequency in patients compared to controls; ▾, decreased frequency in patients compared to controls.

Unsupervised analysis on pre-gated T-cells (CD3+), B-cells (CD19+), and all other cells (CD3-CD19-) using the CAPX pipeline (27) was used to identify 50 T-cell clusters (Supplementary Figure 2), 20 B-cell clusters (Supplementary Figure 3), and 10 non-T non-B clusters (Supplementary Figure 4). Analysis of the data using t-distributed stochastic neighbor embedding (t-SNE) did not demonstrate any apparent differences between patients and controls (Supplementary Figures 2–4). However, analysis using significance analysis of microarrays (SAM) indicated that four T-cell clusters (Figure 4A), four B-cell clusters (Figure 4B), and one non-T non-B cluster (Figure 4C) differed in frequency between burn survivors and controls (SAM test delta adjusted so that the type 1 error rate was 0%).

**Figure 5:**
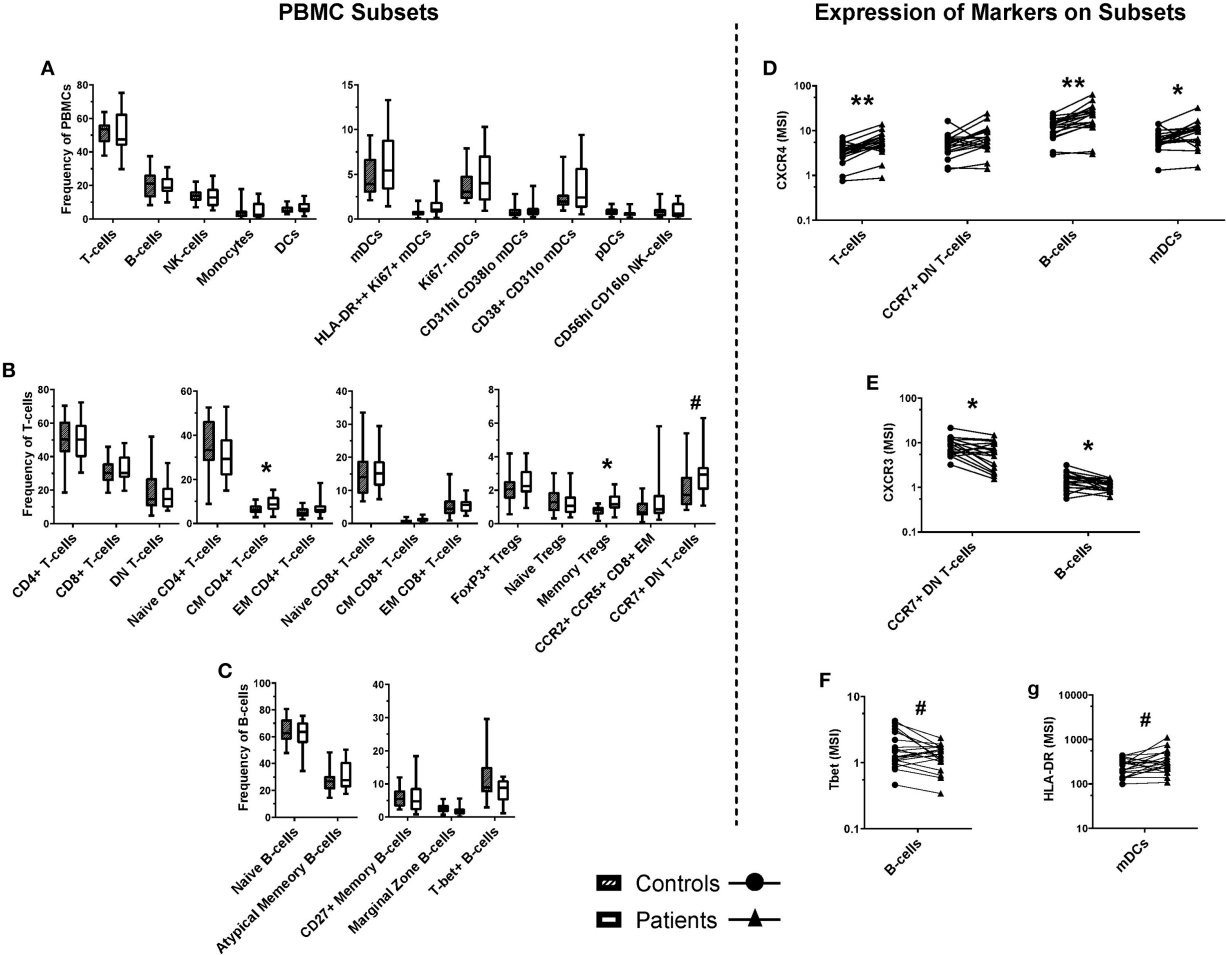
Frequencies of cell populations in pediatric burn patients vs. matched controls, and the expression of markers on several subsets. A binary gating approach was used to manually identify leukocyte subpopulations in PBMCs stained for mass cytometry. Significant differences were identified using Wilcoxin signed-rank test. **(A)** Populations analyzed a frequency of total PBMCs, including overall CD3+ (T-cells) and CD19+ (B-cells), NK cells, monocytes, and dendritic cells. **(B)** T-cell subpopulations analyzed as a frequency of CD3+ cells. Significant increases were identified in the frequency of central memory CD4+ T-cells and memory Tregs, and a trending increase was identified in the frequency of CCR7+ double-negative (CD4- CD8-) T-cells. **(C)** B-cell subpopulations analyzed as a frequency of CD19+ CD20+ cells. Unsupervised analysis informed the investigation of signal intensity for **(D)** CXCR4 on T-cells, B-cells, and mDCs, **(E)** CXCR3 on DN T-cells and B-cells, **(F)** Tbet on B-cells, and **(G)** HLA-DR on mDC subpopulations. CXCR4 expression was found to be increased across T-cells, B-cells, and mDCs, while CXCR3 expression was decreased on CCR7+ DN T-cells and B-cells. A trending decrease in the expression of Tbet was identified in B-cells, and a trending increase in the expression of HLA-DR **p < 0.01, *p < 0.05, ^#^*p* < 0.1. CD, cluster of differentiation; mDC, myeloid dendritic cells; pDC, plasmacytoid dendritic cells; CM, central memory; NK, natural killer; Th, T-helper; DN, double negative (CD4- CD8-), T-bet, T-box expressed in T-cells, CCR, C-C pattern chemokine receptor, CXCR, C-X-C pattern chemokine receptor, IgD, immunoglobulin D. MSI, mean signal intensity. *n* = 20 age/sex-matched controls.

Supervised analysis using a curated gating strategy informed by the unsupervised analysis (whereby markers present on clustered populations were used to focus investigation) identified a difference in the frequencies of several T-cell subpopulations. However, there were no changes in frequency of B-cell, NK, or myeloid cell populations (Figures 5A–C). There was a significant increase in the frequency of central memory (CM) CD4+ T-cells (CD3+ CD4+ CD45RO+ CCR7+; 1.42-fold, *p* < 0.05) in the burn group compared to controls. There were also changes in the frequency of memory T-regulatory cells (32) (Tregs; CD3+ CD4+ CD25+ CD127-low FoxP3+ CD45RO+; 1.69-fold, *p* < 0.05) in the burn group compared to controls. In addition, there was a trend toward a higher frequency of CCR7+ double-negative (CD4- CD8-) T-cells in the burn group that failed to reach statistical significance (1.42-fold *p* ≈ 0.06) (Figure 5B).

Several markers that were expressed by the clusters highlighted by SAM analysis of the FlowSOM data were investigated by geometric mean signal intensity on manually gated subpopulations corresponding with those identified by unsupervised analysis (Figures 5D–G). Interestingly, there was a significant increase in mean expression of the chemokine receptor CXCR4 on T-cells, B-cells, and mDCs in burn survivors compared to controls (1.67-fold, *p* < 0.01; 1.81-fold, *p* < 0.01; and 1.52-fold, *p* < 0.05, respectively). There was also a significant 0.73-fold decrease (*p* < 0.05) in the expression of CXCR3 on B-cells and CCR7+ DN T-cells (33, 34) in burn survivors, and a trend toward lower Tbet expression in B-cells and higher HLA-DR expression by mDCs.

## Discussion

Burns patients have a lifelong increase in the likelihood of developing a range of chronic inflammatory conditions (9–14, 35). The possibility of immune disruption in acute burn injuries, and more specifically severe burn trauma, has been extensively investigated. It has been established that burn injury shifts hematopoiesis to increased production of myeloid cells in the acute response to severe injury (36, 37), and there is a transient increase in circulating DC frequency after the sudden drop seen soon after severe burns (38). In cases involving sepsis, DCs fail to regain normal numbers in the circulation in the weeks following injury. Severe burn injury has also been found to abrogate proinflammatory DC responses and to disrupt DC-mediated T-cell priming, increasing the risk of infection for at least 5 days following injury (39). It is clear that burn injuries, specifically severe burn injuries, result in acute immune dysregulation. Current research has understandably focused on improving survival for those worst impacted by burn trauma. What remains unclear is whether these changes persist, and to what extent they manifest in survivors of non-severe burn injuries.

In this study we performed a comprehensive analysis of immune parameters in children who had suffered a burn to <10% of total body surface area at least 3 years prior, compared to age- and sex-matched controls. We noted multiple abnormalities in the patient cohort, including increased plasma cytokines, decreased vaccine responses, and a number of changes in immune cell populations and the expression of immune molecules by those populations.

Circulating concentrations of TNF-α, IL-7, IL-2, and IFN-γ were all elevated in the patient cohort. DTPa-specific antibodies were lower resulting in diminished rates of seroprotection to diphtheria and pertussis seropositivity amongst the burn survivors. The frequency of memory T-cell subsets—central memory CD4+ T-cells and memory Tregs—was higher for burn survivors, and expression of CXCR4 across B-cells, T-cells, and myeloid dendritic cells was increased. CXCR3 expression on B-cells and a subset of double-negative T-cells was lower in patients than controls. The increase in central memory CD4+ T-cells and memory Tregs is consistent with the findings in a recent report that used mass cytometry to investigate blood immune subsets in complex regional pain syndrome, another condition in which inflammation persists long after the original injury (40).

Elevated plasma levels of TNF-α are associated with inflammation and are a risk factor for cardiovascular disease (41). TNF-α is also implicated in the development of diabetes mellitus and inflammatory polyarthropathies (42–44). A sustained elevation of TNF-α suggests that burn survivors have a chronic inflammatory condition. This would typically be driven by macrophages, which have been shown to persist in scar tissue many weeks after injury (45). However, TNF-α secreting M1 macrophages are generally replaced by M2 macrophages after several weeks of healing (46, 47). An alternative source of TNF-α (and IL-2 and IFN-γ) in the scar/skin microenvironment is tissue resident memory T-cells (Trm) (48). It is unknown whether a distinct population of Trm persists in burn scars or other parts of the dermis, but they have previously been associated with chronic inflammatory diseases and pose an interesting avenue for further research (48).

IL-7 is produced by stromal cells in the bone marrow, thymus, and lymph nodes, all hematopoietic tissues, and cells found in the skin, including keratinocytes and fibroblasts (49, 50). IL-7 is necessary for lymphoid proliferation and maturation, and acts to maintain peripheral homeostasis of T-cells (51). Exposure to IL-7 has been associated with increased expression of CXCR4 in mature T-cells, which may explain the significantly increased expression of CXCR4 on T-cell subsets in burn survivors. IL-7 is known to protect against apoptosis in T-cells via increased expression of Bcl2 (52), which contributes to T-cell survival. This may have an impact on the T-cell frequencies observed in burn survivors, particularly (central) memory T-cells, as IL-7 supports the transition from effector phenotypes to long-term memory (53).

CXCR4 is involved in the regulation of hematopoiesis, bone marrow homing, and sequestering progenitor cells in the bone marrow (54). We did not identify any differences in the frequency of circulating progenitor cells, and there was no significant decrease in the frequency of any PBMC subset despite an increase in CXCR4 expression across B-, T-, and myeloid cells. This study did not consider changes to the bone marrow niche, which may be disrupted by aberrant CXCR4 expression, or the expression of the CXCR4 ligand CXCL12, which is constitutively expressed by bone marrow stromal cells (55).

Dendritic cells have been shown to downregulate MHC-II expression in response to IL-7 (56). However, our data show a trend toward increased expression of HLA-DR in burn survivors at 3 years post-injury. Decreased expression of MHC-II on DCs has been reported in studies using mouse models of burn injury (20) at 3 months post-injury; the duration of this reduction is unknown, though the data presented here suggests it does not persist for 3 years. Elevated IL-7 concentrations could potentially be contributing to low seroprotection rates in burn survivors via DC MHC-II downregulation at the time of vaccination, although further studies will be required to confirm such a mechanism.

IFN-γ is secreted by CD4+ T-cells, CD8+ effector T-cells, macrophages, and NK cells. Although there were no significant differences in the frequencies of CD8+ T-cells or NK cells between burn survivors and controls (macrophages were not assessed in this study) there may have changes in cell phenotype. Potentially there is a skew in CD8+ T cells or NK cells toward cytokine secreting rather than cytotoxic cells that contributes to the elevated levels of IFN-γ in burn survivors. The expression of IFN-γ in response to stimulation was not assessed for this study, but should be considered for future work, along with cytotoxicity markers including Granzyme B and Perforin. The functional responses of these cells *ex vivo* or after stimulations with antigen may highlight changes in the burn survivor's immune system.

Of note, IFN-γ is a regulator of DC maturation (57), which is associated with the increased expression of CXCR4 on plasmacytoid DCs (58, 59), which are potent type I IFN producers. Interestingly in this burn patient cohort we found increased CXCR4 expression on the myeloid DC population. In keeping with recent findings of increased viral infection and cancer in burn patients (11, 14), studies indicate prolonged type I IFN production is linked with immune cell dysfunction in both viral infection and cancer (60). The implications for post-burn pathophysiology are unclear—however increased CXCR4 expression by circulating DCs may reflect an overall increase in DC maturation, particularly in secondary lymphoid tissues where DCs drive T-cell responses through antigen presentation and co-stimulatory activation.

IFN-γ has also been shown to directly upregulate the expression of the immune checkpoint molecule PD-L1 that contributes to immune tolerance (61, 62). It is likely that the long-term increase in IFN-γ titres in burn survivors is associated with an increase in expression of PD-1/PD-L1, similar to the increase in PD-1/PD-L1 across B- and T-cells in patients with sepsis or severe burn injury (63). Indeed, the multiorgan failure that is a major cause of morbidity in sepsis, and is associated with increased risk of infection and ineffective adaptive immunity, may be due to aberrant expression of checkpoint molecules leading to impaired immune responses, particularly in T-cells (64). Similar increases in PD-1/PD-L1 have been observed in patients undergoing surgery with systemic inflammatory response syndrome (65).

T-bet expression in B-cells is associated with antigen experience and IgG2a/c class-switching (66). Alternatively, CXCR3 expression in B-cells is associated with IgG1 co-expression (67), and is involved in lymphoid follicular homing and this may contribute to reduced antibody isotype switching (33). We observed a decrease in CXCR3 expression on B-cells in burn survivors, with a trend for decreased T-bet expression, and this may contribute to the reduced DTPa IgG concentrations, and decreased seroprotection/seropositivity rates. Alternatively, an immunosuppressive environment may drive the difference between T- and B-cell phenotypes in patients and controls: the dominant function of IL-2 is to support the differentiation, survival and function of regulatory T-cells (68). The increased frequency of memory Tregs in burn survivors may reflect increased availability of IL-2. Additionally, IL-2 inhibits the development of T follicular helper cells which have a role in the regulation of B-cell proliferation and class-switching (69, 70). Immunosuppression and tolerance have an important role in the pathogenesis and progression of cancer and infection (15, 71), and this may further contribute to the severity of post-burn morbidities.

There are observed similarities with the changes observed in this study and those observed with severe burn injury and other severe pathologies such as sepsis. Multiorgan failure due to pronounced systemic inflammation is a major cause of morbidity in sepsis, however these patients are also observed to be at increased risk of infection and demonstrate ineffective adaptive immunity (64). Other studies of the impact of burn injury have shown sustained elevated cytokine levels, with IL-1α, Il-7 and IFN-γ all shown to be elevated for up to 1–2 months post-injury in pediatric patients (final follow-up (5, 72). In the long-term, widespread elevation of cytokines has been observed up to 3 years post-severe burn injury in children (21), with only IL-12p70 and MIP1β not showing sustained elevation. These studies also demonstrate long-term clinical impacts of the burn on metabolism and physical function, supporting a likely link of this hyperinflammation to pathology.

Our study did not include PD-1 or PD-L1 in our marker panel, so we cannot draw direct conclusions regarding long-term immune checkpoint dysfunction in survivors of NSBI. However, evidence exists that demonstrates a severe burn injury, in conjunction with bacterial infection, can lead to increased PD-L1 expression in a mouse model, with improved survival at 7 days following anti-PD-L1 therapy (73). Therefore, further studies to examine immune checkpoint in NSBI are warranted.

Our approach provides a broad snapshot of the immune system in pediatric burn survivors. Whilst many of the changes observed were subtle between the two groups, given the epidemiological, patient and animal study evidence for sustained impacts of burn injury (7–14, 20, 21), we believe it is likely these subtle immune changes, magnified over time, contribute to the increase in susceptibility to disease. However, there are limitations to these findings. The scope of the studywas restricted to the circulating components of the immune system and cannot provide any insight into the constituents of different tissues. Therefore, we cannot draw any conclusions regarding differences between patients and controls that may exist in the bone marrow, lymphoid organs, skin, and other tissues—e.g., it may be more informative to investigate immune memory in the bone marrow (74). The relatively small sample size is also a key limitation of this study and further patient recruitment will be important to validate findings from this cohort. Nevertheless, this study is comparable to those of others that have also identified changes in PBMC profiles associated with sustained pathology (40) and provides new insight into the possible consequences of acute burn injury and an important basis for further research. Most importantly, whilst these experiments provide observations of changes in these cell populations, functional assays will be critical to understand the potential clinical consequences of the observed disparity between groups.

In conclusion, this study provides evidence of an enduring change to the circulating components of the immune system in pediatric burn survivors at least 3 years after a non-severe burn injury. Burn survivors appeared to have a more limited response to the DTPa vaccine booster (administered at 4 years of age), and significant changes to T-cell lineages, coupled with disparate expression of surface proteins and transcription factors in T-, B- and dendritic cells. This suggests an ongoing impact of burn trauma on the immune system. These changes hint at a mechanism that may drive the rates of post-burn infections and other diseases controlled by the adaptive immune response. Further work to unravel the link between this disparity and the secondary morbidities observed in burn survivors will be important to understanding the systemic impacts of burn trauma, and the development of therapeutic pathways to reduce the incidence of morbidity in children who recover from a burn.

## Data Availability Statement

The raw data supporting the conclusions of this article will be made available by the authors, without undue reservation. Debarcoded files are uploaded to Flow Repository http://flowrepository.org/id/FR-FCM-Z2XE.

## Ethics Statement

The studies involving human participants were reviewed and approved by Child and Adolescent Health Service Ethics committee. Written informed consent to participate in this study was provided by the participants' legal guardian/next of kin.

## Author Contributions

BJ, HM, AS, ML, FW, MF, and VF: study concept and design. FW, PR, VP, SP, DP, and JM: patient recruitment and clinical expertise. BJ, HM, SM, and VP: experimental work. BJ, HM, ML, MF, and BF: data analysis and interpretation. All authors: manuscript preparation and revision.

## Conflict of Interest

The authors declare that the research was conducted in the absence of any commercial or financial relationships that could be construed as a potential conflict of interest.

## References

[1] Mathers C Fat DM Boerma JT World Health Organization The Global Burden of Disease: 2004 Update. Geneva: World Health Organization (2008).

[2] Tracy L McInnes J Gong J Gabbe B Thomas T BRANZ Annual Report July 2016. Melbourne, VIC (2017). p. 61.

[3] Duke J Wood F Semmens J Spilsbury K Edgar DW Hendrie D. A 26-Year Population-based study of burn injury hospital admissions in Western Australia. J Burn Care Res. (2011) 32:379–86. 10.1097/BCR.0b013e318219d16c 21448072

[4] Brusselaers N Monstrey S Vogelaers D Hoste E Blot S. Severe burn injury in europe: a systematic review of the incidence, etiology, morbidity, and mortality. Crit Care. (2010) 14:R188. 10.1186/cc9300 20958968 PMC3219295

[5] Jeschke MG Chinkes DL Finnerty CC Kulp G Suman OE Norbury WB. The pathophysiologic response to severe burn injury. Ann Surg. (2008) 248:387–401. 10.1097/SLA.0b013e3181856241 18791359 PMC3905467

[6] Gauglitz GG Song J Herndon DN Finnerty CC Boehning D Barral JM. Characterization of the inflammatory response during acute and post-acute phases after severe burn. Shock. (2008) 30:503–7. 10.1097/SHK.0b013e31816e3373 18391855 PMC7863568

[7] Duke JM Randall SM Fear MW O'Halloran E Boyd JH Rea S. Long term cardiovascular impacts after burn and non-burn trauma: a comparative population-based study. Burns. (2017) 43:1662–72. 10.1016/j.burns.2017.08.001 29032972

[8] O'Halloran E Shah A Dembo L Hool L Viola H Grey C. The impact of non-severe burn injury on cardiac function and long-term cardiovascular pathology. Sci Rep. (2016) 6:34650. 10.1038/srep34650 27694999 PMC5046146

[9] Vetrichevvel TP Randall SM Fear MW Wood FM Boyd JH Duke JM. Burn injury and long-term nervous system morbidity: a population-based cohort study. BMJ Open. (2016) 6:e12668. 10.1136/bmjopen-2016-012668 27609857 PMC5020894

[10] Randall SM Fear MW Wood FM Rea S Boyd JH Duke JM. Long-term musculoskeletal morbidity after adult burn injury: a population-based cohort study. BMJ Open. (2015) 5:e009395. 10.1136/bmjopen-2015-009395 26362668 PMC4567662

[11] Duke JM Bauer J Fear MW Rea S Wood FM Boyd J. Burn injury, gender and cancer risk: population-based cohort study using data from Scotland and Western Australia. BMJ Open. (2014) 4:e003845. 10.1136/bmjopen-2013-003845 24441050 PMC3902327

[12] Duke JM Randall SM Fear MW Boyd JH Rea S Wood FM. Diabetes mellitus after injury in burn and non-burned patients: a population based retrospective cohort study. Burns. (2018) 44:566–72. 10.1016/j.burns.2017.10.019 29306596

[13] Stevenson AW Randall SM Boyd JH Wood FM Fear MW Duke JM. Burn leads to long-term elevated admissions to hospital for gastrointestinal disease in a West Australian population based study. Burns. (2017) 43:665–73. 10.1016/j.burns.2016.09.009 27720266

[14] Fear VS Boyd JH Rea S Wood FM Duke JM Fear MW. Burn injury leads to increased long-term susceptibility to respiratory infection in both mouse models and population studies. PLoS ONE. (2017) 12:e0169302. 10.1371/journal.pone.0169302 28068397 PMC5221812

[15] Vinay DS Ryan EP Pawelec G Talib WH Stagg J Elkord E. Immune evasion in cancer: mechanistic basis and therapeutic strategies. Semin Cancer Biol. (2015) 35:S185–98. 10.1016/j.semcancer.2015.03.004 25818339

[16] Allan SM Rothwell NJ. Inflammation in central nervous system injury. Philos Trans R Soc Lond B Biol Sci. (2003) 358:1669–77. 10.1098/rstb.2003.1358 14561325 PMC1693261

[17] Hidalgo E Essex SJ Yeo L Curnow SJ Filer A Cooper MS. The response of T cells to interleukin-6 is differentially regulated by the microenvironment of the rheumatoid synovial fluid and tissue. Arthritis Rheum. (2011) 63:3284–93. 10.1002/art.30570 22038403

[18] Neuman MG. Immune dysfunction in inflammatory bowel disease. Transl Res. (2007) 149:173–86. 10.1016/j.trsl.2006.11.009 17383591

[19] Pickup JC. Inflammation and activated innate immunity in the pathogenesis of type 2 diabetes. Diabetes Care. (2004) 27:813–23. 10.2337/diacare.27.3.813 14988310

[20] Valvis SM Waithman J Wood FM Fear MW Fear VS. The immune response to skin trauma is dependent on the etiology of injury in a mouse model of burn and excision. J Invest Dermatol. (2015) 135:2119–28. 10.1038/jid.2015.123 25826422

[21] Jeschke MG Gauglitz GG Kulp GA Finnerty CC Williams FN Kraft R. Long-term persistence of the pathophysiologic response to severe burn injury. PLoS ONE. (2011) 6:e21245. 10.1371/journal.pone.0021245 21789167 PMC3138751

[22] Xing D Wirsing von König CH Newland P Riffelmann M Meade BD Corbel M. Characterization of reference materials for human antiserum to pertussis antigens by an international collaborative study. Clin Vaccine Immunol. (2009) 16:303–11. 10.1128/CVI.00372-08 19109448 PMC2650870

[23] van Gageldonk PGM van Schaijk FG van der Klis FR Berbers GAM. Development and validation of a multiplex immunoassay for the simultaneous determination of serum antibodies to Bordetella pertussis, diphtheria and tetanus. J Immunol Methods. (2008) 335:79–89. 10.1016/j.jim.2008.02.018 18407287

[24] Kaur S Sehgal R Shastry SM McCaughan G McGuire HM Fazekas de St Groth B. Circulating endothelial progenitor cells present an inflammatory phenotype and function in patients with alcoholic liver cirrhosis. Front Physiol. (2018) 9:556. 10.3389/fphys.2018.00556 29872403 PMC5972283

[25] Saeys Y van Gassen S Lambrecht BN. Computational flow cytometry: helping to make sense of high-dimensional immunology data. Nat Rev Immunol. (2016) 16:449–62. 10.1038/nri.2016.56 27320317

[26] Leipold MD Obermoser G Fenwick C Kleinstuber K Rashidi N McNevin JP. Comparison of CyTOF assays across sites: results of a six-center pilot study. J Immunol Methods. (2018) 453:37–43. 10.1016/j.jim.2017.11.008 29174717 PMC5805584

[27] Ashhurst T Cytometry Analysis Pipeline for Large and CompleX Datasets v2.5. (2018). Available online at: https://github.com/sydneycytometry/CAPX (accessed January 01, 2019).

[28] van Gassen S Callebaut B van Helden MJ Lambrecht BN Demeester P Dhaene T. FlowSOM: using self-organizing maps for visualization and interpretation of cytometry data. Cytometry A. (2015) 87:636–45. 10.1002/cyto.a.22625 25573116

[29] Maecker HT McCoy JP Nussenblatt R. Standardizing immunophenotyping for the human immunology project. Nat Rev Immunol. (2012) 12:191–200. 10.1038/nri3158 22343568 PMC3409649

[30] Saeed A I Sharov V White J Li J Liang W Bhagabati N. TM4: a free, open-source system for microarray data management and analysis. BioTechniques. (2003) 34:374–8. 10.2144/03342mt01 12613259

[31] Tusher VG Tibshirani R Chu G. Significance analysis of microarrays applied to the ionizing radiation response. PNAS. (2001) 98:5116–21. 10.1073/pnas.091062498 11309499 PMC33173

[32] Rosenblum MD Way SS Abbas AK. Regulatory T cell memory. Nat Rev Immunol. (2016) 16:90–101. 10.1038/nri.2015.1 26688349 PMC5113825

[33] D'Acquisto F Crompton T CD3+CD4-CD8- (double negative) T cells: saviours or villains of the immune response? Biochem Pharmacol. (2011) 82:333–40. 10.1016/j.bcp.2011.05.019 21640713

[34] Haug T Aigner M Peuser MM Strobl CD Hildner K Mougiakakos D. Human double-negative regulatory T-cells induce a metabolic and functional switch in effector T-cells by suppressing mTOR activity. Front Immunol. (2019) 10:883. 10.3389/fimmu.2019.00883 31105702 PMC6498403

[35] Barrett LW Fear VS Waithman JC Wood FM Fear MW. Understanding acute burn injury as a chronic disease. Burns Trauma. (2019) 7:23. 10.1186/s41038-019-0163-2 31534977 PMC6745803

[36] Posluszny JA Muthumalaiappan K Kini AR Szilagyi A He L-K Li Y. Burn injury dampens erythroid cell production through reprioritizing bone marrow hematopoietic response. J Trauma. (2011) 71:1288–96. 10.1097/TA.0b013e31822e2803 22071930 PMC3217199

[37] Johnson NB Posluszny JA He LK Szilagyi A Gamelli RL Shankar R. Perturbed MafB/GATA1 axis after burn trauma bares the potential mechanism for immune suppression and anemia of critical illness. J Leukoc Biol. (2016) 100:725–36. 10.1189/jlb.1A0815-377R 26992433 PMC6608024

[38] D'Arpa N Accardo-Palumbo A Amato G D'Amelio L Pileri D Cataldo V. Circulating dendritic cells following burn. Burns. (2009) 35:513–8. 10.1016/j.burns.2008.05.027 19269101

[39] Shen H de Almeida PE Kang KH Yao P Chan CW. Burn injury triggered dysfunction in dendritic cell response to TLR9 activation and resulted in skewed T cell functions. PLoS One. (2012) 7:e50238. 10.1371/journal.pone.0050238 23189191 PMC3506591

[40] Russo MA Fiore NT van Vreden C Bailey D Santarelli DM McGuire HM Expansion and activation of distinct central memory T lymphocyte subsets in complex regional pain syndrome. J Neuroinflammation. (2019) 16:63 10.1186/s12974-019-1449-9 30885223 PMC6423749

[41] Zhang H Park Y Wu J Chen XP Lee S Yang J. Role of TNF-α in vascular dysfunction. Clin Sci. (2009) 116:219–30. 10.1042/CS20080196 19118493 PMC2620341

[42] Akash MSH Rehman K Liaqat A. Tumor necrosis factor-alpha: role in development of insulin resistance and pathogenesis of type 2 diabetes mellitus. J Cell Biochem. (2018) 119:105–10. 10.1002/jcb.26174 28569437

[43] Li P Schwarz EM. The TNF-α transgenic mouse model of inflammatory arthritis. Springer Semin Immunopathol. (2003) 25:19–33. 10.1007/s00281-003-0125-3 12904889

[44] Vasanthi P Nalini G Rajasekhar G Role of tumor necrosis factor-alpha in rheumatoid arthritis: a review: TNF-α in RA. APLAR J Rheumatol. (2007) 10:270–4. 10.1111/j.1479-8077.2007.00305.x

[45] Tarran S Langlois NEI Dziewulski P Sztynda T. Using the inflammatory cell infiltrate to estimate the age of human burn wounds: a review and immunohistochemical study. Med Sci Law. (2006) 46:115–26. 10.1258/rsmmsl.46.2.115 16683466

[46] Parisi L Gini E Baci D Tremolati M Fanuli M Bassani B Macrophage polarization in chronic inflammatory diseases: killers or builders? J Immunol Res. (2018) 2018:115–26. 10.1155/2018/8917804 PMC5821995 29507865

[47] Chen L Wang J Li S Yu Z Liu B Song B. The clinical dynamic changes of macrophage phenotype and function in different stages of human wound healing and hypertrophic scar formation. Int Wound J. (2019) 16:360–9. 10.1111/iwj.13041 30440110 PMC7948805

[48] Steinbach K Vincenti I Merkler D Resident-memory T cells in tissue-restricted immune responses: for better or worse? Front Immunol. (2018) 9:2827 10.3389/fimmu.2018.02827 30555489 PMC6284001

[49] Yamanaka K–I Clark R Rich B Dowgiert R Hirahara K Hurwitz D. Skin-derived interleukin-7 contributes to the proliferation of lymphocytes in cutaneous T-cell lymphoma. Blood. (2006) 107:2440–5. 10.1182/blood-2005-03-1139 16322477 PMC1895734

[50] Hara T Shitara S Imai K Miyachi H Kitano S Yao H. Identification of IL-7-Producing cells in primary and secondary lymphoid organs using IL-7-GFP knock-in mice. J Immunol. (2012) 189:1577–84. 10.4049/jimmunol.1200586 22786774

[51] Gao J Zhao L Wan Y Zhu B. Mechanism of action of IL-7 and its potential applications and limitations in cancer immunotherapy. Int J Mol Sci. (2015) 16:10267–80. 10.3390/ijms160510267 25955647 PMC4463645

[52] Wykes M Macpherson G. Dendritic cell-B-cell interaction: dendritic cells provide B cells with CD40-independent proliferation signals and CD40-dependent survival signals. Immunology. (2000) 100:1–3. 10.1046/j.1365-2567.2000.00044.x 10809952 PMC2326988

[53] Li J Huston G Swain SL. IL-7 promotes the transition of CD4 effectors to persistent memory cells. J Exp Med. (2003) 198:1807–15. 10.1084/jem.20030725 14676295 PMC2194161

[54] Burger JA Bürkle A. The CXCR4 chemokine receptor in acute and chronic leukaemia: a marrow homing receptor and potential therapeutic target. Br J Haematol. (2007) 137:288–96. 10.1111/j.1365-2141.2007.06590.x 17456052

[55] Janssens R Struyf S Proost P. The unique structural and functional features of CXCL12. Cell Mol Immunol. (2018) 15:299–311. 10.1038/cmi.2017.107 29082918 PMC6052832

[56] Guimond M Veenstra RG Grindler DJ Zhang H Cui Y Murphy RD. Interleukin 7 signaling in dendritic cells regulates the homeostatic proliferation and niche size of CD4+ T cells. Nat Immunol. (2009) 10:149–57. 10.1038/ni.1695 19136960 PMC2713006

[57] Pan J Zhang M Wang J Wang Q Xia D Sun W. Interferon-γ is an autocrine mediator for dendritic cell maturation. Immunol Lett. (2004) 94:141–51. 10.1016/j.imlet.2004.05.003 15234546

[58] Lee B Sharron M Montaner LJ Weissman D Doms RW. Quantification of CD4, CCR5, and CXCR4 levels on lymphocyte subsets, dendritic cells, and differentially conditioned monocyte-derived macrophages. Proc Natl Acad Sci USA. (1999) 96:5215–20. 10.1073/pnas.96.9.5215 10220446 PMC21844

[59] McKenna K Beignon A-S Bhardwaj N. Plasmacytoid dendritic cells: linking innate and adaptive immunity. J Virol. (2005) 79:17–27. 10.1128/JVI.79.1.17-27.2005 15596797 PMC538703

[60] Snell LM McGaha TL Brooks DG. Type I interferon in chronic virus infection and cancer. Trends Immunol. (2017) 38:542–57. 10.1016/j.it.2017.05.005 28579323 PMC8059441

[61] RoŽman P Švajger U. The tolerogenic role of IFN-γ. Cytokine Growth Factor Rev. (2018) 41:40–53. 10.1016/j.cytogfr.2018.04.001 29655565

[62] Zhang X Zeng Y Qu Q Zhu J Liu Z Ning W. PD-L1 induced by IFN-γ from tumor-associated macrophages via the JAK/STAT3 and PI3K/AKT signaling pathways promoted progression of lung cancer. Int J Clin Oncol. (2017) 22:1026–33. 10.1007/s10147-017-1161-7 28748356

[63] Wilson JK Zhao Y Singer M Spencer J Shankar-Hari M. Lymphocyte subset expression and serum concentrations of PD-1/PD-L1 in sepsis - pilot study. Crit Care. (2018) 22:95. 10.1186/s13054-018-2020-2 29661225 PMC5902875

[64] Patil NK Guo Y Luan L Sherwood ER. Targeting immune cell checkpoints during sepsis. Int J Mol Sci. (2017) 18:2413. 10.3390/ijms18112413 29135922 PMC5713381

[65] Monaghan SF Thakkar RK Tran ML Huang X Cioffi WG Ayala A. Programmed death 1 expression as a marker for immune and physiological dysfunction in the critically Ill surgical patient. Shock. (2012) 38:117–22. 10.1097/SHK.0b013e31825de6a3 22683728

[66] Myles A Gearhart PJ Cancro MP. Signals that drive T-bet expression in B cells. Cell Immunol. (2017) 321:3–7. 10.1016/j.cellimm.2017.09.004 28923237 PMC6191851

[67] Muehlinghaus G Cigliano L Huehn S Peddinghaus A Leyendeckers H Hauser AE. Regulation of CXCR3 and CXCR4 expression during terminal differentiation of memory B cells into plasma cells. Blood. (2005) 105:3965–71. 10.1182/blood-2004-08-2992 15687242

[68] Malek TR. The main function of IL-2 is to promote the development of T regulatory cells. J Leukoc Biol. (2003) 74:961–5. 10.1189/jlb.0603272 12960253

[69] Liao W Lin J-X Leonard WJ. Interleukin-2 at the crossroads of effector responses, tolerance, and immunotherapy. Immunity. (2013) 38:13–25. 10.1016/j.immuni.2013.01.004 23352221 PMC3610532

[70] Pallikkuth S de Armas L Rinaldi S Pahwa S. T follicular helper cells and B cell dysfunction in aging and HIV-1 infection. Front Immunol. (2017) 8:1380. 10.3389/fimmu.2017.01380 29109730 PMC5660291

[71] George MP Masur H Norris KA Palmer SM Clancy CJ McDyer JF. Infections in the immunosuppressed host. Ann Am Thorac Soc. (2014) 11:S211–20. 10.1513/AnnalsATS.201401-038PL 25148427 PMC4200572

[72] Finnerty CC Jeschke MG Herndon DN Gamelli R Gibran N Klein M. Temporal cytokine profiles in severely burned patients: a comparison of adults and children. Mol Med. (2008) 14:553–60. 10.2119/2007-00132.Finnerty 18548133 PMC2424320

[73] Patil NK Luan L Bohannon JK Hernandez A Guo Y Sherwood ER. Frontline science: anti-PD-L1 protects against infection with common bacterial pathogens after burn injury. J Leukoc Biol. (2018) 103:23–33. 10.1002/JLB.5HI0917-360R 29345058 PMC9680647

[74] Chang H Tokoyoda K Radbruch A. Immunological memories of the bone marrow. Immunol Rev. (2018) 283:86–98. 10.1111/imr.12656 29664564 PMC5947123

