# Corrigendum: Pediatric Burn Survivors Have Long-Term Immune Dysfunction With Diminished Vaccine Response

**DOI:** 10.3389/fimmu.2020.598646

**Published:** 2020-10-09

**Authors:** Blair Z. Johnson, Sonia McAlister, Helen M. McGuire, Vetrichevvel Palanivelu, Andrew Stevenson, Peter Richmond, Debra J. Palmer, Jessica Metcalfe, Susan L. Prescott, Fiona M. Wood, Barbara Fazekas de St Groth, Matthew D. Linden, Mark W. Fear, Vanessa S. Fear

**Affiliations:** ^1^School of Biomedical Sciences, The University of Western Australia, Perth, WA, Australia; ^2^School of Medicine, The University of Western Australia, Perth, WA, Australia; ^3^Wesfarmers Centre of Vaccines and Infectious Diseases, Telethon Kids Institute, Perth, WA, Australia; ^4^Ramaciotti Facility for Human Systems Biology and the Charles Perkins Centre, Discipline of Pathology, The University of Sydney, Sydney, NSW, Australia; ^5^Centre for Allergy and Immunology Research, Telethon Kids Institute, Perth, WA, Australia; ^6^Department of Health WA, Perth, WA, Australia; ^7^Genetic and Rare Diseases, Telethon Kids Institute, Perth, WA, Australia

**Keywords:** non-severe burn injury, immunity, vaccination, mass cytometry, acute trauma, systemic

In the original article, there was an error in the URL provided in Data Availability Statement. The updated statement appears below.

The authors apologize for this error and state that this does not change the scientific conclusions of the article in any way. The original article has been updated.

## Data Availability Statement

The raw data supporting the conclusions of this article will be made available by the authors, without undue reservation. Debarcoded files are uploaded to Flow Repository http://flowrepository.org/id/FR-FCM-Z2XE.

