# Supplementary material for: Pediatric Burn Survivors Have Long-Term Immune Dysfunction With Diminished Vaccine Response
Source: Front Immunol. 2020 Jul 21;11:1481. doi: 10.3389/fimmu.2020.01481 (PMC7385079; doi:10.3389/fimmu.2020.01481)
Supplement: Supplementary file 1 [file Data_Sheet_1.docx]

**Supplementary Data**
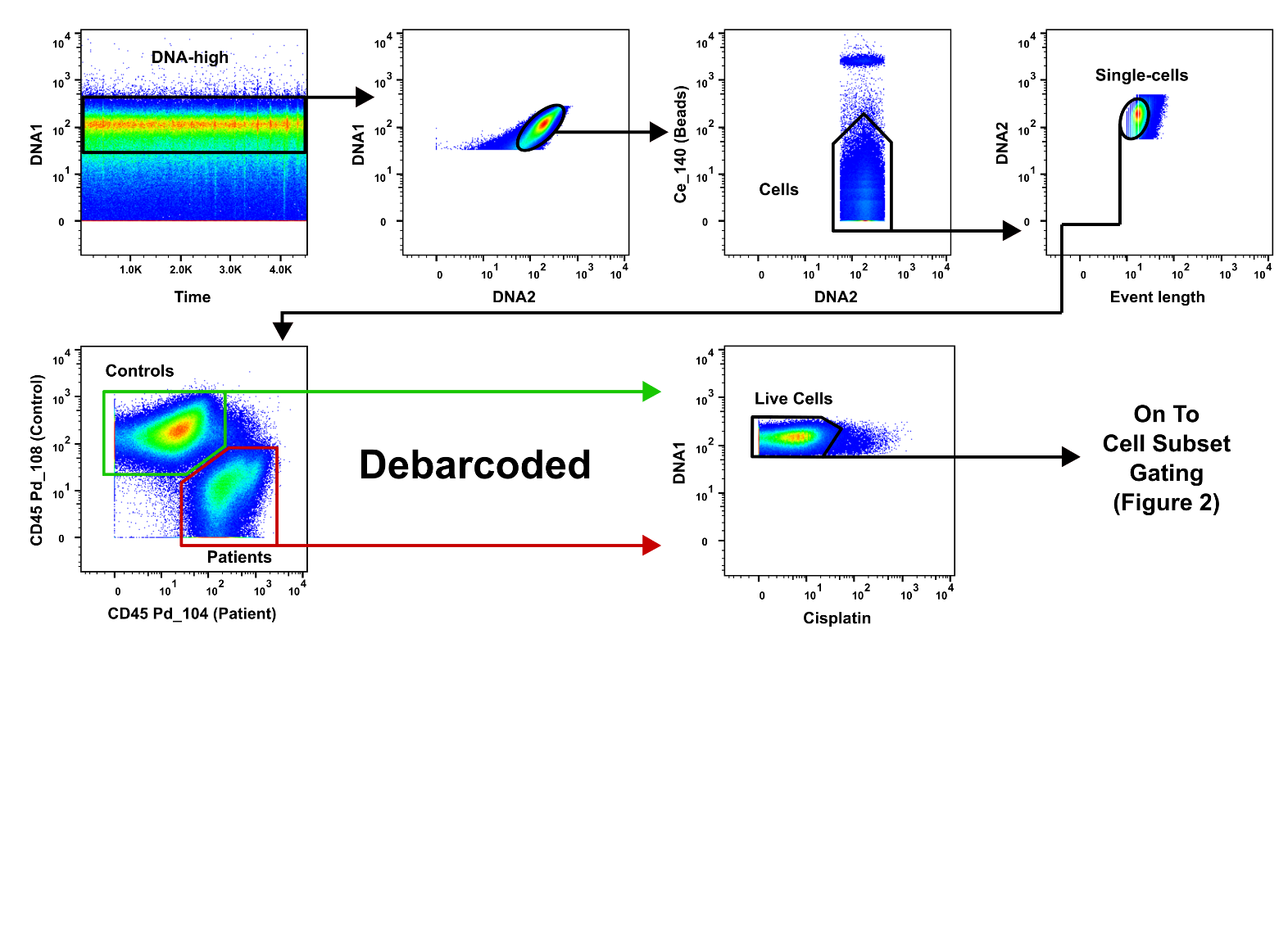


**Supplementary Figure 1. Clean-up gating and debarcoding.** DNA1_Ir191 and DNA2_Ir193 for detecting isotopes of DNA intercalator. Live cells are gated on debarcoded samples separately.


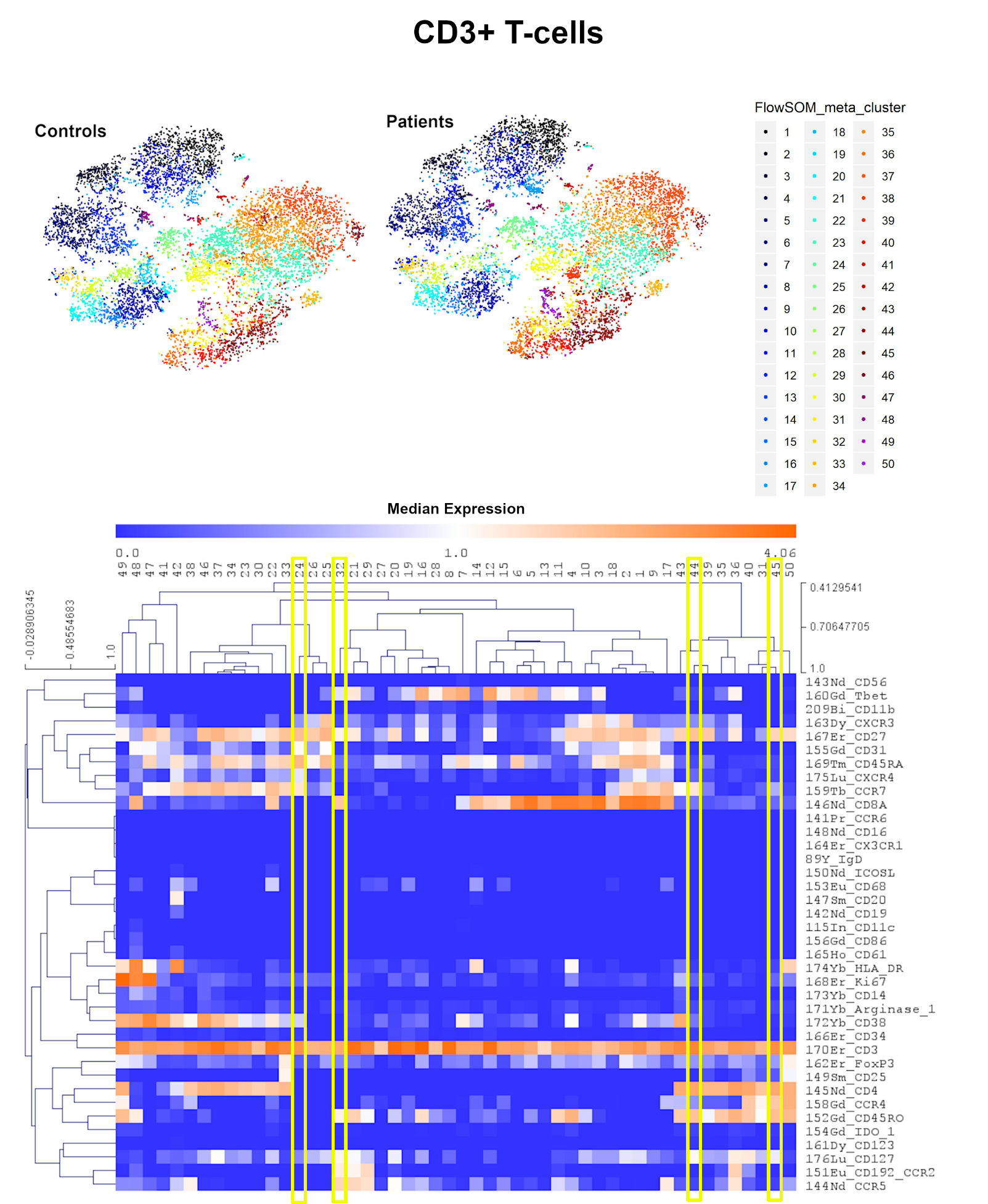


**Supplementary Figure 2. tSNE output and expression mapping of FlowSOM clusters of CD3+ events (T-cells).** Patient and control events were clustered and tSNE calculated in one file, then tSNE graphs of each were subsequently produced for comparison. Expression plot shows the median expression of events in each cluster for each marker. Clusters with different frequencies between patients and controls (SAM analysis) are highlighted in yellow. Events were clustered using all markers in each file except CD45, DNA-intercalator, cisplatin, and CD61. n = 20 age/sex-matched controls.


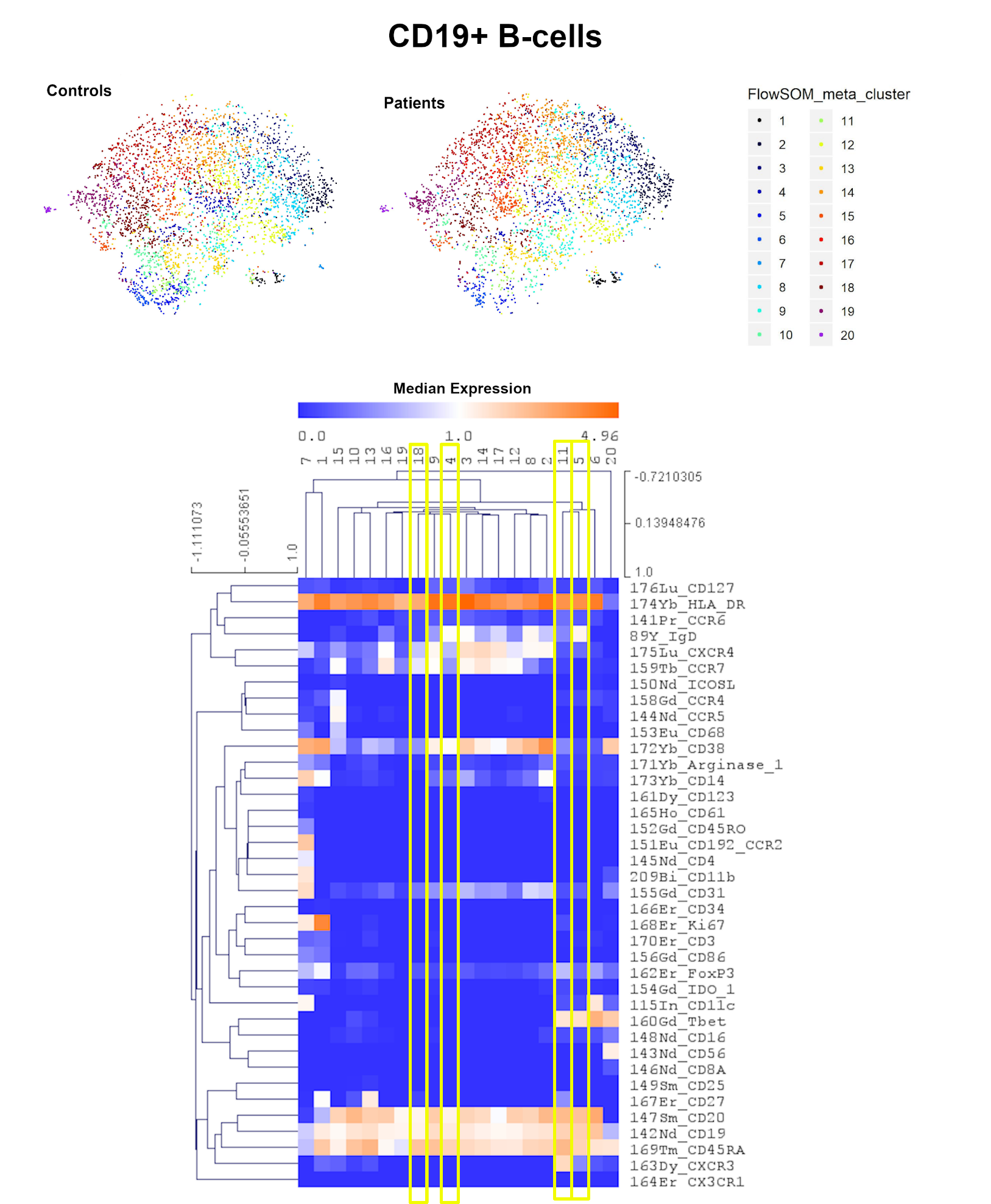


**Supplementary Figure 3. tSNE output and expression mapping of FlowSOM clusters of CD19+ events (B-cells).** Patient and control events were clustered and tSNE calculated in one file, then tSNE graphs of each were subsequently produced for comparison. Expression plot shows the median expression of events in each cluster for each marker. Clusters with different frequencies between patients and controls (SAM analysis) are highlighted in yellow. Events were clustered using all markers in each file except CD45, DNA-intercalator, cisplatin, and CD61. n = 20 age/sex-matched controls.


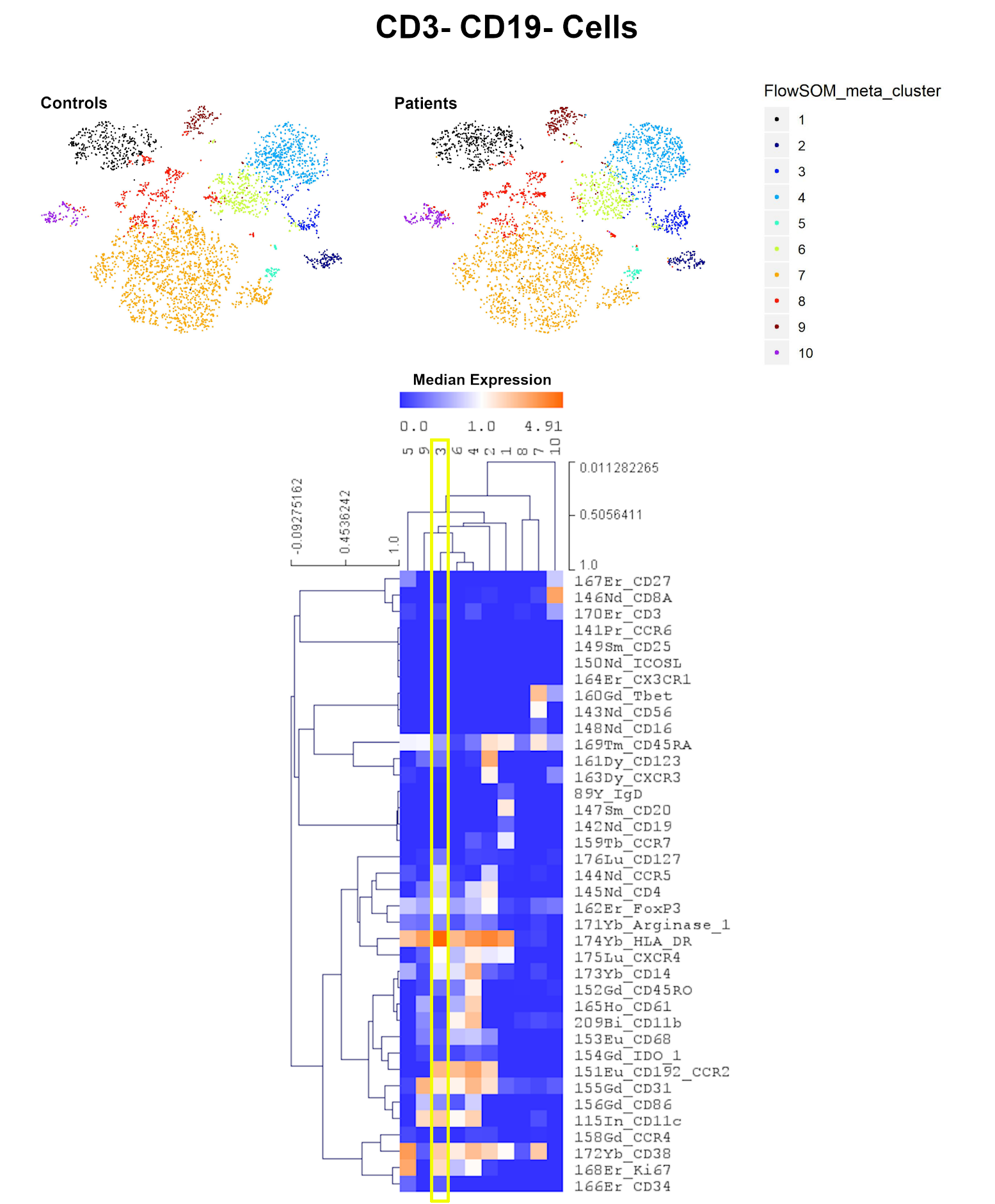


**Supplementary Figure 4. tSNE output and expression mapping of FlowSOM clusters of CD3- CD19- events.** Patient and control events were clustered and tSNE calculated in one file, then tSNE graphs of each were subsequently produced for comparison. Expression plot shows the median expression of events in each cluster for each marker. Clusters with different frequencies between patients and controls (SAM analysis) are highlighted in yellow. Events were clustered using all markers in each file except CD45, DNA-intercalator, cisplatin, and CD61. n = 20 age/sex-matched controls.
